# Use of Tannate of Alumina

**Published:** 1852-02

**Authors:** 


*Use of Tannate of Alumina.*—Mr. Harrison had found the local exhibition of the remedy in question followed by the most satisfactory results. The method of using was to throw into the passage an injection containing from 2 to 10 grains of the salt dissolved in distilled water, the strength of the solution being in a great measure determined by the amount of smarting pain produced. The most advisable method was just to keep the strength of the injection up to the smarting point. He thought it injurious to produce more than a gentle scalding.

Mr. Harrison did not anticipate, of course, equal success in every case, but he generally found the disordered condition of the urethral mucous membrane removed in the course of one or two weeks, in the ordinary run of cases.

On his recommendation, some of his professional friends had employed it in their practice, and from their reports he was supported in his high opinion of the remedial properties of the tannate of alumina. The combination of alumina and tannic acid produced by Mr. Rogers Harrison, was of a dirty yellowish color, and in crystals about the size of those of coarse sugar, and readily soluble in hot water.—*Lon. Med. Gaz.*

